# Supplementary material for: Quantitative analysis of Weibel-Palade bodies
Source: PLoS One. 2022 Dec 21;17(12):e0278044. doi: 10.1371/journal.pone.0278044 (PMC9770420; doi:10.1371/journal.pone.0278044)
Supplement: S1 File — (DOCX) [file pone.0278044.s001.docx]

fileName=File.nameWithoutExtension;

setSlice(1); //start with DAPI

run("Enhance Contrast", "saturated=0.35");

run("Apply LUT", "slice");

run("Set Scale...", "distance=1")

run("8-bit")

run("Gaussian Blur...", "sigma=5 slice")

run("Convert to Mask", "method=Huang background=Light only");

run("Set Measurements...", "area mean min display redirect=None decimal=3");

run("Analyze Particles...", "size=1000-Infinity show=Masks display summarize add in_situ slice");

selectWindow("Results");

saveAs("Results", "/Volumes/Seagate External SSD/WPB/2020_06_08/HUVECs/24hr/Results_DAPI/ " + fileName + "_DAPI.csv");

selectWindow("Results");

run("Close");

setSlice(2); //switches to FITC

run("Enhance Contrast", "saturated=0.35");

run("Apply LUT", "slice");

run("Set Scale...", "distance=1")

run("8-bit")

run("Convert to Mask", "method=Huang background=Light only");

run("Set Measurements...", "area mean min display redirect=None decimal=3");

run("Analyze Particles...", "size=10-infinity circularity=0.05-0.8 show=Masks display summarize in_situ slice");

selectWindow("Results");

saveAs("Results", "/Volumes/Seagate External SSD/WPB/2020_06_08/HUVECs/24hr/Results_FITC/ " + fileName + "_FITC.csv");

selectWindow("Results");

run("Close");
