# Supplementary material for: Quantitative analysis of Weibel-Palade bodies
Source: PLoS One. 2022 Dec 21;17(12):e0278044. doi: 10.1371/journal.pone.0278044 (PMC9770420; doi:10.1371/journal.pone.0278044)
Supplement: Attachment — Submitted filename: reviewers_comments.docx [file pone.0278044.s002.docx]

- a clear hypothesis and justification of the study is needed, especially, that WBP density in cultured endothelial cells is not t new idea and topic. What does the study add to the field in terms of new knowledge?

**Answer**

We thank the reviewer for highlighting this and we have corrected it. We have updated the introduction, line 63, with the addition of a clear hypothesis.

- Please clearly indicate the number of biological parallels as well as the number of independent experiments.

**Answer**

We agree with the reviewer, we did not clearly state the number of biological parallels and number of independent experiments conducted. We have corrected this by clarifying in the methods section, line 232 and have ensured that each figure states the appropriate number of replicates.

- add individual data points to figures (in addition to the mean)

**Answer**

We thank the reviewer for bringing to our attention how to improve our figures. Individual data points have been added to each figure, in addition to the mean.

- junctional staining should be also performed to clearly show cell morphology

**Answer**

We thank the reviewer for the suggestion of junctional staining to clearly show the cell morphology. Unfortunately, due to the fact our slides are permanently fixed, we are unable to add additional staining. As such, we have put in our Results and Discussion that the lack of junctional staining may limit further analysis. In future studies, we will consider including junctional staining.

Please include the following items when submitting your revised manuscript:

- A rebuttal letter that responds to each point raised by the academic editor and reviewer(s). You should upload this letter as a separate file labeled 'Response to Reviewers'.
- A marked-up copy of your manuscript that highlights changes made to the original version. You should upload this as a separate file labeled 'Revised Manuscript with Track Changes'.
- An unmarked version of your revised paper without tracked changes. You should upload this as a separate file labeled 'Manuscript'.

**Comments to the Author**

1. Is the manuscript technically sound, and do the data support the conclusions?

The manuscript must describe a technically sound piece of scientific research with data that supports the conclusions. Experiments must have been conducted rigorously, with appropriate controls, replication, and sample sizes. The conclusions must be drawn appropriately based on the data presented.

Reviewer #1: Yes

Reviewer #2: Partly

**Answer**

We thank reviewer #2 for highlighting the lack in clarification of controls, replication, and sample size. We have addressed this concern and updated the methods section, line 232, to state the clear independent experiments and biological parallels.

2. Has the statistical analysis been performed appropriately and rigorously?

Reviewer #1: Yes

Reviewer #2: Yes

3. Have the authors made all data underlying the findings in their manuscript fully available?

The [PLOS Data policy](http://www.plosone.org/static/policies.action#sharing) requires authors to make all data underlying the findings described in their manuscript fully available without restriction, with rare exception (please refer to the Data Availability Statement in the manuscript PDF file). The data should be provided as part of the manuscript or its supporting information, or deposited to a public repository. For example, in addition to summary statistics, the data points behind means, medians and variance measures should be available. If there are restrictions on publicly sharing data—e.g. participant privacy or use of data from a third party—those must be specified.

Reviewer #1: Yes

Reviewer #2: No

**Answer**

We thank Reviewer #2 for bringing to our attention the lack of clarification in the statistics and data points. We have addressed this by adding individual data points to our figures and updating the statistics in figure descriptions. In addition, we have added a data sharing agreement statement.

4. Is the manuscript presented in an intelligible fashion and written in standard English?

PLOS ONE does not copyedit accepted manuscripts, so the language in submitted articles must be clear, correct, and unambiguous. Any typographical or grammatical errors should be corrected at revision, so please note any specific errors here.

Reviewer #1: Yes

Reviewer #2: Yes

5. Review Comments to the Author

Please use the space provided to explain your answers to the questions above. You may also include additional comments for the author, including concerns about dual publication, research ethics, or publication ethics. (Please upload your review as an attachment if it exceeds 20,000 characters)

Reviewer #1: Lui et al have investigated how endothelial cells reaching confluence determines their WPB load. Using HUVECs and ECFCs they find that endothelial cells, when given the opportunity to form confluent monolayers will reach some sort of a plateau in terms of WPB numbers per cell. This confirms data from Howell et al from >15 years ago. Furthermore, they show that the route taken to in vitro confluence, be it higher initial seeding density or prolonged incubation time, has hardly any influence on the WPB plateau. This paper lacks a clear, biologically relevant hypothesis or concept to put the results of these experiments into context or warrant their publication. As such, the value of this paper is very limited.

**Answer**

We thank Reviewer #1 for their concern of a lack of value for this paper. We demonstrate that the method of reaching confluency does not matter in terms of quality and quantity of WPB. Though this is a valid concern, we believe that our manuscript offers detailed analysis for endothelial cell culture which provides researchers a reference to more efficiently plan and execute experiments.

Minor comments:
Figure 1: The images shown are very grainy. The typical elongated morphology of WPBs is difficult to discern and large clusters of granules stacked on top of on another appear to present. With this kind of detail I am not sure how accurate the quantifications are. Is this a PDF conversion issue?

**Answer**

We thank the reviewer for bringing to our attention the quality of our images to best convey the details in WPB morphology. Due to the upload format for PLOS, we submitted images inserted into a document. We will work with the editorial team for options to provide full resolution image files to resolve this concern. All quantifications/analysis were done on the full-resolution images.

Reviewer #2: The article describes the effect of confluency on WPB numbers in endothelial cells (both HUVEC and ECFCs). The authors compare WPB number in cells seeded at differing densities and cells allowed to reach confluency over a differing period of time.

The effect of confluency on WPB number has been known for a while but there have been contradictory publications which have somewhat muddied the water and this manuscript provides clarity, examines different endothelial types and different ways to get confluent cells and is therefore worthy.

Generally the results are displayed appropriately and the experiments appear sound. Summary data is shown throughout, showing individual experimental points in addition would make the results easier to interpret. More detail in the legends would also help. N numbers are mentioned in the methods but they should also be included in the legends as well as a description of what the error bars represent (SD, SEM etc). Similarly when the WPB numbers are quoted in the text the SD or SEM should also be included to allow readers to assess the veracity of the data.

**Answer**

We thank Reviewer #2 for the meticulous review and we have updated our legends and descriptions.

Fig.1
The legend on the scale bar is really small and should be easier to read. Error bars represent SEM/SD? It would be helpful to have an image at 0h to compare to 100h. It us unclear which timepoint has been shown.

**Answer**

We thank the reviewers for brining to our attention the lack of specificity in our images. We have updated the descriptions to clarify what timepoint the images were taken. We recognize that the scale bar is really small and will work with the editorial team to provide higher resolution images. Unfortunately, we do not have images taken at 0 hours, as this is when we seed the cells. As the process of suspending, counting, and seeding cells can alter their physiology, we chose to not image at time = 0 hours. We have added a t = 4 hour image for comparison.

Fig.2
Summary data alone is shown. Individual experimental points would help interpretation (or this data should be shown as supplementary) B and D need error bars ideally.

**Answer**

We thank the reviewers for pointing out the lack of detail of individual data points. Instead of summary data, we have included all individual experimental points. We have added error bars to_______

Fig.3 and 4
Summary data alone is shown, individual data points need to be shown also. The definition of confluency is arbitrary, the authors need to give some idea as to how they delineated confluent vs non confluent with representative images. Microscopy of proteins present at the junctions such as VE Cadherin would make this clearer and allow the readers to gauge the effects and compare the cell types (the authors mentioned differences in cell size etc).

**Answer**

We thank the reviewers for comments on the definition of confluency. We agree that the definition of confluency can be arbitrary and can vary in different laboratories and approaches. For our study, we defined it as a qualitative assessment coverage of 80% of an immunofluorescent image. It should be noted that once our approach was defined, it was applied universally across the analysis. For example, the cutoffs of 51 cells/field for HUVECs and 75 cells/field for ECFCs were universally applied across all images to define “confluent” and “non-confluent” images to minimize bias. It is not the case that each image was individually assessed qualitatively to be “confluent” vs “non-confluent.” We have added multiple revisions to make this point more clear, beginning in line 257. As noted in a response to reviewer 1, We thank the reviewer for the suggestion of junctional staining to clearly show the cell morphology. Unfortunately, due to the fact our slides are permanently fixed, we are unable to add additional staining. As such, we have put in our Results and Discussion that the lack of junctional staining may limit further analysis. In future studies, we will consider including junctional staining.

6. PLOS authors have the option to publish the peer review history of their article ([what does this mean?](https://journals.plos.org/plosone/s/editorial-and-peer-review-process#loc-peer-review-history)). If published, this will include your full peer review and any attached files.


If you choose “no”, your identity will remain anonymous but your review may still be made public.


**Do you want your identity to be public for this peer review?** For information about this choice, including consent withdrawal, please see our [Privacy Policy](https://www.plos.org/privacy-policy).

Reviewer #1: No

Reviewer #2: No
